# Comparative study of thyroid puncture biopsy guided by contrast-enhanced ultrasonography and conventional ultrasound

**DOI:** 10.3892/etm.2013.1016

**Published:** 2013-03-15

**Authors:** FENGSHENG LI, HUITING LUO

**Affiliations:** 1 Department of Ultrasound, Shaanxi Provincial Cancer Hospital Affiliated to Medical School, Xi’an Jiaotong University, Xi’an, Shaanxi 710061;; 2 Department of Ultrasound, Affiliated Hospital of Shaanxi University of Chinese Medicine, Xi’an, Shaanxi 712000, P.R. China

**Keywords:** contrast-enhanced ultrasound, ultrasound, papillary thyroid carcinoma, puncture biopsy

## Abstract

The aim of this study was to investigate the application value of thyroid puncture biopsy guided by contrast-enhanced ultrasound (CEUS). A total of 48 patients with 51 solid thyroid nodules (suspected papillary thyroid carcinoma, PTC) were enrolled in the study. Following detection by conventional ultrasonography and CEUS, puncture biopsy of the suspicious lesions guided by conventional ultrasonography and CEUS was conducted, respectively. Then, pathological diagnosis was performed. The number of PTC positive nodules and puncture points detected by the two methods were compared. In 51 nodules with 310 punctures, 44 nodules (86.3%, 44/51) and 240 punctures (77.4%, 240/310) were pathologically diagnosed as PTC. In the 44 nodules diagnosed as PTC, 43 and 34 nodules were detected by CEUS and conventional ultrasound, respectively, with a significant difference between the two methods (P=0.022). Eleven (25%) nodules were independently detected by CEUS. The sensitivity and accuracy of puncture point detection by CEUS (82.9 and 82.6%, respectively) were significantly higher compared with those of conventional ultra-sound (48.3 and 56.5%, respectively; P<0.001). The specificity of puncture points detected by CEUS (81.4%) was significantly lower compared with that by conventional ultrasound (84.3%; P=0.009). Compared with conventional ultrasound, a greater number of PTC-positive nodules were detected by CEUS, with increased sensitivity and accuracy of the puncture points.

## Introduction

Due to excellent spatial and temporal resolution, ultrasound has become the first choice for the imaging examination of thyroid nodules, with a detection rate as high as 70% (1,2). The incidence of adult thyroid nodules is 20–75%, among which 5–6.5% of nodules are diagnosed as malignant (3,4). Thyroid disease is complex and varied and the differentiation between benign and malignant nodules is clinically difficult. Fine needle aspiration biopsy (FNAB) guided by ultrasound is the gold standard for the diagnosis of thyroid cancer; however, the dissatisfaction rate of tissue drawing is ∼10–20% (5). Therefore, an effective imaging technique based on optimized systematical puncture biopsy to accurately determine the position of the thyroid lesions is required. This is likely to improve the detection rate of thyroid cancer, reduce unnecessary repeated biopsies and reduce the incidence of complications.

As identified in previous studies, the contrast-enhanced ultrasound (CEUS) technique improves the detection rate of lesions and diagnosis sensitivity (6–8). Bartolotta *et al*(9) performed CEUS on 18 patients with a solitary thyroid nodule and confirmed its feasibility. The second generation CEUS contrast agent SonoVue contains sulfur hexafluoride microbubbles, which generate resonance under the action of an acoustic wave with low mechanical index. The low acoustic energy emission and pulse inversion harmonic imaging are combined to clearly exhibit the microvascular perfusion in tumors (10). CEUS has been successfully applied in the diagnosis and interventional therapy of focal liver lesions. Ultrasound guidance effectively improves the safety of aspiration biopsy. However, due to the difficulties in discriminating between necrotic and non-necrotic areas in a tumor with conventional ultrasound, the positive rate of biopsies is low, with increased numbers of false negative results. In the current study, CEUS combined with microflow imaging (MFI) was applied to guide the puncture biopsy of the thyroid, and compared with conventional ultrasound. The application value of CEUS is also discussed.

## Subjects and methods

### Subjects

A total of 48 patients with 51 suspected solid thyroid nodules in Shaanxi Provincial Cancer Hospital Affiliated to Medical School, Xi’an Jiaotong University from May 2011 to September 2012 were enrolled in this study. There were 13 males with 13 nodules and 35 females with 38 nodules. They were aged 17–64 years, with an average age of 47.2±5.7 years. The diameter of the nodules was 0.6–4.3 cm (average, 1.6 cm). Patients hypersusceptible to SonoVue or with coagulation dysfunction were excluded. This study was approved by the ethics committee of Shaanxi Provincial Cancer Hospital Affiliated to Medical School, Xi’an Jiaotong University. Informed consent was obtained from all patients.

### Apparatus and methods

An iU22 ultrasound machine (L9-3 probe; Philips Medical Systems, Ltd., Eindhoven, The Netherlands) was used to detect the thyroid. The normal thyroid tissue around the nodule was selected as the contrast cross-section and puncture cross-section. The suspicious solid thyroid nodule was used as the inclusion criterion, with no background of diffuse thyroid lesions. A microbubble suspension prepared with 59 mg SonoVue dry powder (Bracco Imaging S.p.A Inc., Milan, Italy) and 5 ml normal saline was used as the contrast agent. An intravenous injection in the elbow with 2.4 ml contrast agent with low mechanical index (0.06–0.08) was administered and the process of contrast agent infusion in the tumor was observed.

Patients lay in the supine position, with their neck on a high pad. Following conventional disinfection, local infiltration anesthesia with 2% lidocaine was conducted. A BARD^®^ automatic biopsy gun (Bard Biopsy Systems, Tempe, AZ, USA) combined with an 18G biopsy needle was used for the biopsy. The puncture point and needle insertion angle were determined. The puncture direction and position were monitored by ultrasound and the non-enhancement area with liquefaction necrosis was avoided. When the biopsy needle reached the tumor margin and the ejection distance and direction were determined, the tissues in the tumor marginal area and high-enhancement area were directly drawn, followed by pathological diagnosis by senior pathologists. The whole process was conducted under strictly aseptic conditions. Following biopsy, local gentle pressing was conducted for hemostasis.

### Statistical analysis

Statistical analysis was performed using SPSS 13.0 statistical software (SPSS Inc., Chicago, IL, USA). McNemar’s test was used to compare the positive number of nodules and puncture point positive rate between CEUS and conventional ultrasound. The Chi-square test was performed to compare the positive and negative puncture point predictive values between the two methods. P<0.05 was considered to indicate a statistically significant difference.

## Results

### Number of PTC nodules detected by the two methods

**Figure 1:**
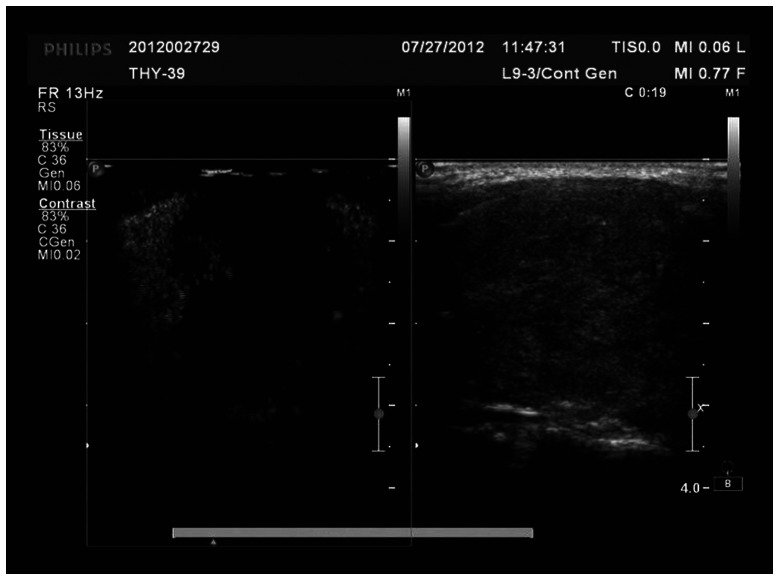
Irregular weak concentric ring enhancement by CEUS (pathologically diagnosed as PTC). CEUS, contrast-enhanced ultrasonography; PTC, papillary thyroid carcinoma.

**Figure 2:**
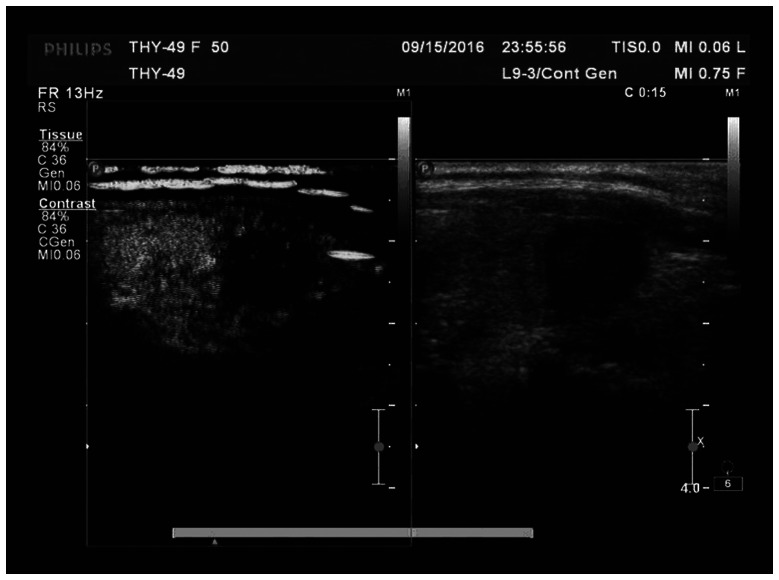
Weak enhancement by CEUS (pathologically diagnosed as PTC). CEUS, contrast-enhanced ultrasonography; PTC, papillary thyroid carcinoma.

**Figure 3:**
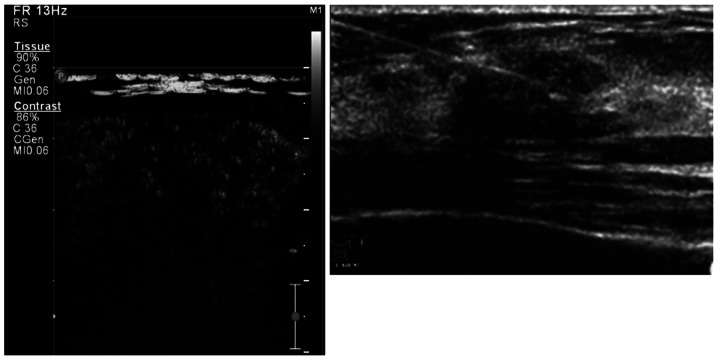
Uneven enhancement by CEUS (pathologically diagnosed as PTC). CEUS, contrast-enhanced ultrasonography; PTC, papillary thyroid carcinoma.

There were three types of manifestation, including irregular weak concentric ring enhancement (Fig. 1), no or weak enhancement (Fig. 2) and uneven enhancement (Fig. 3) in the CEUS images of the 51 nodules in 48 patients. All nodules provided adequate specimens and the satisfaction rate of tissue drawing was 100%. Of the 51 nodules, 44 (86.3%), five (9.8%) and two (3.9%) nodules were pathologically diagnosed as PTC, nodular goiter and focal Hashimoto’s disease, respectively. From the 44 nodules diagnosed as PTC, 43 (97.7%) and 34 (77.3%) nodules were detected by CEUS and conventional ultrasound, respectively, with a significant difference between the two methods (P=0.022). Eleven (25%) nodules were independently detected by CEUS and 31 (70.5%) nodules were detected by CEUS and conventional ultrasound. Only one nodule was not detected (2.3%).

### Positive detection rates of puncture points detected by the two methods

In 310 puncture points for 51 nodules, there were 240 (77.4%), 56 (18.1%) and 14 (4.5%) puncture points that were pathologically diagnosed as PTC, nodular goiter and focal Hashimoto’s disease, respectively. There were 127 and 212 puncture points detected by conventional ultrasound and CEUS, with 2.49 and 4.16 puncture points per patient, respectively. The number of detected puncture points and the specificity and accuracy of the puncture points detected by CEUS and conventional ultrasound were significantly different.

The pathological findings of the puncture points for the two methods are shown Table I. In the 240 puncture points pathologically diagnosed as PTC, 116 (48.3%) and 199 (82.9%) puncture points were detected by conventional ultrasound and CEUS, respectively. In the 310 puncture points, the 175 (56.5%) and 256 (82.6%) puncture points detected by conventional ultrasound and CEUS, respectively, were consistent with pathological findings. The sensitivity and accuracy of the puncture point detection by CEUS were higher than those by conventional ultrasound, respectively (P<0.001). The specificity of puncture points detected by CEUS was lower than that by conventional ultrasound (P=0.009). The negative predictive values of puncture points detected by CEUS and conventional ultrasound were 88.9% and 76.9%, respectively, with a significant difference (P<0.001; Table II).

## Discussion

The incidence of PTC is increasing annually (11), which may be related to new detection means and increased awareness of PTC (12,13). Although the prognosis of PTC is good, lymph node metastasis and a high recurrence rate may also occur in the early stage (14–16). Therefore, the early determination of nodule nature is clinically significant. The conventional ultra-sound-guided biopsy shows goiter, peripheral vessels and other important structures. However, it is difficult to distinguish the necrotic and non-necrotic areas in the tumor, which results in a low positive rate of tissue drawing. Therefore, methods to improve the positive rate of puncture for thyroid cancer and reduce the complications has become a topic of concern for a number of scholars.

CEUS reflects the locus of contrast agent microbubbles in the tissue vessel, describes the angioarchitecture by a maximum intensity capture technique and clearly shows the microvascular changes that color Doppler flow imaging (CDFI) is not able to show. Therefore, the low-speed small blood vessels in thyroid cancer are better reflected and detected. The neoplastic vessels are usually distributed in a marginal area with active tumor growth. In the central area of the tumor, tumor cells and stroma hyperplasia cause an increase in intratumoral pressure, leading to vascular compression, reduced perfusion, thrombus formation, venous disorders and necrosis (17). In the current study, CEUS-guided puncture biopsy in the high-enhancement area and peripheral area with rich blood supply was performed on 51 nodules and the satisfaction rate of tissue drawing was 100%. In the 44 patients pathologically diagnosed with PTC, 43 cases were detected by CEUS, indicating the clear advantages of CEUS compared with conventional ultrasound. In the 11 nodules independently detected by CEUS, nine nodules exhibited only a low echo by conventional ultrasound; therefore, they were difficult to differentiate from other benign nodules. The sensitivity and accuracy of the puncture points obtained with CEUS are significantly higher than those obtained with conventional ultrasound, which effectively improves the positive rate and accuracy of puncture biopsy for thyroid cancer. This indicates that CEUS is more helpful for selecting suspicious lesions for thyroid puncture biopsy, compared with conventional ultrasound. The results in this study are also consistent with those of previous studies which have reported that CEUS improves the sensitivity of puncture point biopsy (7,8).

The increased positive rate of puncture point biopsy contributes to the detection of a greater number of thyroid cancer patients using fewer puncture points, particularly for cystic-solid mixed nodules and nodules with no clear substantial boundary, regular shape or complete capsule, which conventional ultrasound does not differentiate. CEUS-guided biopsy effectively prevents tumor misdiagnosis and avoids unnecessary repeated sampling for the nodule necrotic area without enhancement. Additionally, it prevents excessive surgical treatment on benign nodules. In this study, 2.49 puncture points per patient were detected by conventional ultrasound. The detection rate was 77.3% (34/44), with 10 missed nodules. For CEUS, 4.16 puncture points per patient were detected. The detection rate was 97.7% (43/44), with only one missed nodule. CEUS detects the majority of malignant nodules, with the advantages of optimum puncture positioning and relatively few puncture points; therefore, it improves the efficiency of targeted puncture biopsy. With the development of CEUS technology, the individualized biopsy scheme with limited puncture points for thyroid tumor will be achieved.

In CEUS-guided thyroid puncture biopsy, multi-time and multi-angle tissue drawing sampling should be performed to improve the sensitivity and accuracy (18,19) and guarantee adequate specimens. Quinn *et al*(20) identified that the diagnosis rate of biopsy using a coarse needle for thyroid disease is significantly higher than that using a fine needle. However, the optimum puncture position and number of puncture points should be determined according to the lesion location and peripheral vital structures. An increase in the number of puncture points is likely to cause repeated sampling and the occurrence of complications. Therefore, the most reliable diagnosis results should be obtained with the fewest puncture points. However, CEUS also has certain limitations: i) for nodules close to the thyroid edge (adjacent to the common carotid artery or capsule), the procedure is highly difficult with a risk of puncture. In these cases, particular attention should be paid to the occurrence of complications. On the basis of local infiltration anesthesia, 3–4 ml saline may be injected into the thyroid and peripheral tissue to establish an isolation belt. This effectively reduces the risk of puncture. ii) For nodules with a diameter <0.5 cm, as the ejection distance of the puncture gun is 1.5–2 cm, the puncture depth should be ∼0.7 cm away from the nodule. The whole needle tract is revealed by ultrasound to enable the nodule to be positioned at the extension line of the needle tract. This is likely to improve the puncture accuracy for a tiny nodule.

CEUS has a higher positive detection rate for thyroid cancer than conventional ultrasound, with higher sensitivity and puncture point accuracy. It has great clinical application value in the guidance of thyroid puncture biopsy and is the preferred method for detecting disease under non-surgical conditions.

## Figures and Tables

**Table I:** Pathological findings of puncture points using the two methods.

		Conventional ultrasound		CEUS	
Pathological finding	Number of puncture points	Positive	Negative	Positive	Negative
PTC positive	240	116	124	199	41
PTC negative	70	11	59	13	57
Total	310	127	183	212	98

CEUS, contrast-enhanced ultrasonography; PTC, papillary thyroid carcinoma.

**Table II:** Comparison of the positive rate of puncture points between the two methods (%).

Method	Sensitivity	Specificity	Accuracy	Positive predictive value	Negative predictive value
Conventional ultrasound	48.3	84.3	56.5	60.5	76.9
CEUS	82.9^a^	81.4^a^	82.6^a^	67.5	88.9^a^

a P<0.05 compared with conventional ultrasound. CEUS, contrast-enhanced ultrasonography.
